# Utility of sentinel surveillance as an early warning system for emerging pathogens in a low-resource setting: Correlations of dengue, chikungunya, Zika, and COVID-19 trends between sentinel and passive surveillance systems in Puerto Rico, 2012–2023

**DOI:** 10.1371/journal.pgph.0006285

**Published:** 2026-06-12

**Authors:** Alfonso C. Hernandez-Romieu, Zachary J. Madewell, Mark Delorey, Hannah R. Volkman, Liliana Sanchez-Gonzalez, Vanessa Rivera-Amill, Diego Sainz de la Peña, Jorge Bertrán-Pasarell, Verónica M. Frasqueri-Quintana, Jomil Torres Aponte, Melissa Marzan-Rodríguez, Aidsa Rivera, Olga Lorenzi, Carla P. Espinet-Crespo, Yashira Maldonado, Roberta Lugo-Robles, Gilberto A. Santiago, Janice Perez-Padilla, Jorge L. Muñoz-Jordán, Gabriela Paz-Bailey, Laura E. Adams

**Affiliations:** 1 Division of Vector-Borne Diseases, Centers for Disease Control and Prevention, San Juan, Puerto Rico; 2 Division of Vector-Borne Diseases, Centers for Disease Control and Prevention, Fort Collins, Colorado, United States of America; 3 Ponce Health Sciences University/Ponce Research Institute, Ponce, Puerto Rico; 4 Auxilio Mutuo Hospital, San Juan, Puerto Rico; 5 Division of Epidemiology and Research, Puerto Rico Department of Health, San Juan, Puerto Rico; Universite de Montreal, CANADA

## Abstract

The representativeness and timeliness of sentinel surveillance for endemic and emerging arboviral and respiratory diseases in low-resource settings are understudied. We compared laboratory-confirmed epidemic dengue, non-epidemic dengue, Zika, chikungunya, and COVID-19 (pre-Omicron and Omicron periods) cases reported in Puerto Rico’s Sentinel Enhanced Dengue Surveillance System (SEDSS) with island-wide trends reported by the Department of Health’s passive disease surveillance system (PADSS). We plotted trends over time to assess representativeness and used lagged cross-correlations to determine whether SEDSS reporting preceded PADSS. SEDSS trends were representative of island-wide trends for all pathogens. SEDSS preceded reporting in PADSS by up to three, eight, and two weeks for epidemic dengue, Zika, and pre-Omicron COVID-19, respectively. Increasing case trends for chikungunya occurred at broadly similar times in both systems, while temporal concordance was lower for non-epidemic dengue. In Puerto Rico, sentinel surveillance was representative of island-wide trends and could provide early warning for dengue epidemics and emerging diseases, such as Zika and COVID-19.

## Introduction

The COVID-19 pandemic and expansion of dengue and chikungunya in the Americas highlight the need to improve surveillance for emerging diseases [1,2]. Arboviral and respiratory viral pathogens have unpredictable and rapid transmission dynamics, requiring surveillance systems that can accurately quantify cases, identify changes in trends in real time, and provide early warning of increasing transmission [3–12]. Many low-resource settings rely on passive surveillance that is limited by case underreporting, incomplete laboratory confirmation, inconsistent epidemiologic and clinical data, and reporting delays [6,13]. Although more resource intensive, sentinel surveillance systems can be implemented in key locations and may be more sensitive in detecting emerging and endemic arboviral and respiratory pathogens.

Sentinel infectious disease surveillance systems are designed to detect emerging pathogens, characterize epidemic trends, and serve as early warning systems in public health. Unlike passive surveillance, sentinel surveillance systems leverage systematic testing of a defined population to detect pathogens more quickly. Because of systematic testing, these systems are not reliant on clinical suspicion to trigger diagnostic testing and are not subject to receipt of information from multiple reporting entities, both of which can limit the completeness and timeliness of passive surveillance test results. Sentinel surveillance systems have proven effective in detecting and characterizing outbreaks of dengue, malaria, HIV/AIDS, and other infectious causes of acute febrile and respiratory illnesses [14–17]. There are limited data, however, regarding the use of sentinel surveillance to obtain representative and timely estimates of emerging arboviral and respiratory pathogen transmission [18–21].

We compared Puerto Rico’s Sentinel Enhanced Dengue Surveillance System (SEDSS) and the Puerto Rico Department of Health (PRDH) passive disease surveillance system (PADSS) to better understand the timeliness and representativeness of sentinel surveillance vis-à-vis passive surveillance for confirmed dengue, chikungunya, Zika, and COVID-19 cases reported during 2012–2023 [22,23]. Our objectives were to 1) determine the representativeness of trends reported in SEDSS compared to island-wide trends reported in PADSS, and 2) examine whether SEDSS could act as an early warning system by identifying trends in cases before PADSS. Our analyses can inform whether a sentinel surveillance system like SEDSS can serve as a reliable and timely surveillance proxy for estimating pathogen transmission trends in settings where under-resourced public health systems may face compounding challenges from disproportionate disease burden.

## Materials and methods

### Ethics statement

The SEDSS protocol was approved by the institutional review boards at the CDC, Auxilio Mutuo Hospital, and Ponce Medical School Foundation (protocol numbers 6214 and 120308-VR). Written informed consent to participate was obtained from all adult participants and emancipated minors. For minors aged 14–20 years, written consent was obtained; for children aged 7–13 years, parental written consent and participant assent were obtained.

### Study design

We compared cases of laboratory-confirmed epidemic dengue (defined as periods with high transmission rates, 2012–2014), non-epidemic dengue (defined as periods with lower or sporadic transmission rates, 2019–2023), Zika (2016–2017), chikungunya (2014–2015), and COVID-19 (during pre-Omicron and Omicron periods) between SEDSS and PADSS. We did not include 2015–2018 in the non-epidemic dengue period due to very low case counts (total n = 237 for the 4 years) that were insufficient to provide meaningful insights into dengue virus transmission patterns.

### Sentinel enhanced dengue surveillance system (SEDSS)

Patients with acute febrile and respiratory illnesses presenting at emergency departments (ED) and outpatient clinics in Puerto Rico are offered enrollment in SEDSS [22,24,25]. SEDSS operated primarily from the Ponce region during 2012–2017 and expanded to include the San Juan Metro Area in 2018 [22]. SEDSS began on May 7, 2012, at Saint Luke’s Episcopal Hospital in Ponce (San Lucas Ponce), a 427-bed tertiary care hospital that receives ~60,000 patients/year and is the regional pediatric referral hospital for the Ponce and Mayaguez (~500,000 residents) Health Districts; enrollment at this site has continued through the present. The Outpatient Acute Care Clinic (CEMI) in Ponce, which provides services to ~20,000 patients/year, began recruiting SEDSS participants on April 1, 2016, and continues through the present. Saint Luke’s Guayama (now Guayama Menonita Hospital), a 161-bed secondary care hospital that receives ~35,000 patients per year from southeastern Puerto Rico (~50,000 residents), enrolled SEDSS participants from February 1, 2013, through September 30, 2015. The Carolina hospital site enrolled participants from July 29, 2013, through September 30, 2015. Hospital Auxilio Mutuo (HAM), a 500-bed tertiary care hospital serving the San Juan Metro Area with ~40,000 emergency room visits and ~14,500 admissions annually, began SEDSS recruitment on September 5, 2018, and continues through the present. For this analysis, we included SEDSS participants who were prospectively enrolled between May 7, 2012, and December 31, 2023.

Patients reporting fever at presentation or in the last 7 days were offered enrollment in SEDSS. Patients could participate once per 14-day period. During the Zika epidemic (June 2016–June 2018), patients with rash, arthritis, arthralgia, or conjunctivitis were eligible regardless of fever. Starting in April 2020, patients with cough or shortness of breath in the last 14 days (with or without fever) were also eligible. Participant serum specimens were tested for dengue virus (DENV) 1–4 (from 2012), chikungunya virus (CHIKV) (from May 2014), and Zika virus (ZIKV) (from November 2015) using reverse transcription-polymerase chain reaction (RT-PCR) and IgM antibody capture (MAC)-ELISA. Before March 2016, DENV was detected using the CDC DENV-1–4 RT-PCR assay [26]. From March 2016–April 2020, the CDC Trioplex RT-PCR assay was used to detect DENV, CHIKV, and ZIKV simultaneously [27], with DENV serotype confirmed using the CDC DENV-1–4 RT-PCR assay. After April 2020, the CDC DENV-1–4 RT-PCR assay was again used as the primary test for DENV. Participants also provided a nasopharyngeal swab for molecular testing for SARS-CoV-2 (from March 2020), influenza A and B, respiratory syncytial virus, parainfluenza 1–4, rhinovirus, human metapneumovirus, and human adenovirus by RT-PCR (from 2012) [17,28].

SEDSS enrolls a relatively consistent number of participants per week, with annual averages ranging from 35 participants per week in 2012–121 participants per week in 2019. Across all years, the overall weekly average is approximately 80 participants (median: 74, IQR: 53–103). Weekly enrollment variability is primarily influenced by the number of eligible patients presenting to care during enrollment hours and staffing levels. The median participation rate ranges from 25–33% in tertiary hospitals and 45–52% in the urgent care clinic. Drop-out rates are < 1%. General metrics on participant eligibility, recruitment, and testing for SEDSS sites during 2012–2022 are detailed elsewhere [22].

### Puerto Rico Department of Health (PRDH) passive surveillance system (PADSS)

As mandated by territorial and federal regulations, all positive results from DENV, CHIKV, and ZIKV RT-PCR and IgM antibody tests, and SARS-CoV-2 RT-PCR and antigen tests conducted in Puerto Rico during the study period had to be reported to PADSS. Suspected infections from symptomatic cases were reported to PADSS based on clinical suspicion, followed by laboratory confirmation. PADSS offers public access to de-identified, individualized test and outcome data for COVID-19 through a web-based dashboard and data repository. For this analysis, we obtained PADSS data extracts for dengue, chikungunya, Zika, and COVID-19 on March 18, 2024. All data provided to the investigators were de-identified prior to receipt; authors did not have access to direct personal identifiers (e.g., names, exact addresses, or medical record numbers).

### Definitions

For both PADSS and SEDSS, epidemic dengue cases were identified by the positive detection of DENV by PCR or IgM tests during January 1, 2012 – December 31, 2014. Non-epidemic dengue cases included positive PCR results or IgM positive results for DENV during January 1, 2019 – December 31, 2023. We considered IgM results for ZIKV where available in addition to positive dengue IgM results, to account for notable cross-reactivity between dengue IgM and Zika IgM antibodies in serologic tests. Cases with positive DENV IgM results but without ZIKV IgM testing after Zika IgM testing ceased in April 2020 were also included in the analysis, acknowledging the unavailability of ZIKV IgM testing during that period.

CHIKV cases were defined as those with positive tests during May 28, 2014 – July 31, 2015. ZIKV cases were those from January 21, 2016, through March 23, 2017. Chikungunya and Zika cases were defined by positive PCR or IgM test results. Zika IgM included negative IgM results for DENV. We restricted analyses of CHIKV and ZIKV to the time periods when the viruses were first detected by either surveillance system through dates covering 95% of the cases identified during the study period. The later stages of the outbreaks were excluded from our analysis to provide a more focused assessment of the surveillance systems’ effectiveness in detecting cases during the peak epidemic phases.

COVID-19 cases in SEDSS are defined as any SEDSS participant who tested positive for SARS-CoV-2 by RT-PCR during a single illness event during March 8, 2020–February 28, 2022. Cases prior to November 28, 2021 (i.e., the date the first case of the Omicron variant was reported in Puerto Rico) were categorized as pre-Omicron, while cases on or after this date were categorized as Omicron. We stratified into pre-Omicron and Omicron periods due to an increase in SARS-CoV-2 testing through PADSS at the emergence of the Omicron variant. SEDSS cases and tests for all pathogens, which are reported to PADSS, were excluded from case and test counts to prevent double counting of individual illness events.

### Statistical analysis

We described frequencies of laboratory confirmed cases of epidemic (2012–2014) and non-epidemic (2019–2023) dengue, chikungunya (2014–2015), Zika (2016–2017), and COVID-19 (2020–2022) by surveillance system, health region, and type of laboratory test. We calculated medians and interquartile ranges (IQR) from symptom onset to specimen collection and from specimen collection to testing by virus and surveillance system. Comparisons between SEDSS and PADSS within each pathogen-period were performed using Pearson chi-square tests for categorical variables, including laboratory test type and overall health region distribution, and Wilcoxon rank-sum tests for timing variables, including symptom onset to specimen collection and specimen collection to PCR or IgM testing.

To visually assess the representativeness of SEDSS relative to PADSS, we plotted weekly case counts of DENV, CHIKV, ZIKV, and COVID-19 by symptom onset week. For descriptive comparison of temporal patterns between surveillance systems, the observed weekly series were smoothed using a centered 3-week moving average. To emphasize temporal pattern rather than case volume, smoothed weekly counts were standardized within each pathogen-period and surveillance system and plotted as weekly share of cases. We used symptom onset week rather than specimen collection date because it more accurately reflects the timing of pathogen transmission.

To examine whether SEDSS could act as an early warning system by identifying trends in cases before PADSS, we lagged the raw weekly case counts by symptom onset week from SEDSS relative to PADSS by ±12 weeks and evaluated the cross-correlation function (CCF) at each lag. No smoothing was applied before the CCF analysis. We used the CCF to determine the lag at which SEDSS cases were most strongly correlated with PADSS at week 0 (i.e., the information available to public health authorities when monitoring case trends). A CCF approaching 1.0 indicates high agreement. For example, if the highest CCF was observed at week -2 in SEDSS relative to PADSS, this would indicate that case reports in SEDSS preceded PADSS by two weeks thus offering some advance warning. To make this determination less empirical, models were fit to SEDSS and PADSS weekly case numbers using the tscount package and we computed Anscombe residuals for both models. We then drew a bootstrap sample from the residuals from each of the two sets, and we paired the residual time series for further analysis. We next transformed these residual series back to the original (observed) scale and computed the CCF and the differences in the CCF between lags. We repeated this 5,000 times using the 2.5^th^ and 97.5^th^ percentiles of these replicates for confidence bounds. This allowed us to infer with some confidence whether the value of the CCF at a given lag was statistically higher from the value of the CCF at another lag. This was done for each virus period (DENV 2012–2014, DENV 2019–2023, CHIKV 2014–2015, ZIKV 2016–2017, SARS-CoV-2 2020–2022). Because SARS-CoV-2 antigen tests were not performed in SEDSS, only RT-PCR tests were used to calculate cross-correlations between SEDSS and PADSS. All analyses were done using R software, version 4.4.1 (R Foundation for Statistical Computing, Vienna, Austria).

## Results

### Cases of dengue, chikungunya, Zika, and COVID-19 in SEDSS and PADSS

Numbers of cases, including their geographic distribution, and median times from symptom onset to specimen collection and from specimen collection to testing for all pathogens are shown in Table 1. Large differences were observed in the median time from specimen collection to PCR testing between the two systems, with SEDSS providing significantly faster turn-around times for all studied pathogens, except for COVID-19, for which PADSS times from specimen collection to testing were not available. The median time from specimen collection to RT-PCR testing by pathogen in SEDSS and PADSS, respectively, were 11 and 15 days for epidemic dengue, 7 and 8 days for non-epidemic dengue, 68 and 166 days for chikungunya, and 4 and 14 days for Zika (P-values ≤0.01). Median times were also significantly lower in SEDSS compared to PADSS for IgM testing for epidemic dengue (9 vs 14 days) and Zika (12 and 17 days) (P-values ≤0.01). SEDSS cases showed geographic variation across pathogen-periods, reflecting the locations of active sentinel sites: epidemic dengue, chikungunya, Zika, and Omicron-period COVID-19 cases were concentrated in the Ponce region, whereas non-epidemic dengue and pre-Omicron COVID-19 cases were more commonly reported from the San Juan health region. PADSS cases for non-COVID pathogens were more geographically dispersed, including substantial proportions reported from the San Juan Metro Area.

**Table 1:** Laboratory-confirmed cases, assay type, timing measures *(median days, IQR)*, and health region distribution *(n, %)* for DENV in epidemic and non-epidemic periods, CHIKV, ZIKV, and COVID-19 reported in SEDSS and PADSS, Puerto Rico, United States.

	DENV epidemic period (2012–2014)		DENV non-epidemic period (2019–2023)		CHIKV (2014–2015)		ZIKV (2016–2017)		COVID-19 pre-Omicron (2020–2021)		COVID-19 Omicron (2022)	
	SEDSS	PADSS	SEDSS	PADSS	SEDSS	PADSS	SEDSS	PADSS	SEDSS	PADSS	SEDSS	PADSS
**Cases and laboratory test type**												
Laboratory-confirmed cases, total, n*	1126	16164	285	3234	2303	4650	1916	34995	544	188728	238	284601
Positive by PCR, n (%)	738 (65.5)	11120 (68.8)	280 (98.2)	3083 (95.3)	**1810 (78.6)**	**4417 (95.0)**	**1448 (75.6)**	**33764 (96.5)**	**544 (100)**	**155223 (82.2)**	**238 (100)**	**173433 (60.9)**
Positive by IgM, n (%)^†^	**854 (75.8)**	**7511 (46.5)**	**89 (31.2)**	**1931 (59.7)**	**889 (38.6)**	**358 (7.7)**	**1255 (65.5)**	**3509 (10.0)**	NA	NA	NA	NA
Positive by SARS-CoV-2 antigen, n (%)	NA	NA	NA	NA	NA	NA	NA	NA	0 (0)	33505 (17.8)	0 (0)	111168 (39.1)
**Timing measures, median days (IQR)** ^**‡**^												
Symptom onset to specimen collection	**3 (1, 4)**	**4 (3, 5)**	**3 (2, 4)**	**4 (2, 5)**	1 (1, 2)	1 (1, 2)	2 (1, 3)	2 (1, 4)	2 (1, 3)	NA	2 (1, 3)	NA
Specimen collection to PCR testing	**11 (8, 16)**	**15 (10, 26)**	**7 (5, 10)**	**8 (5, 16)**	**68 (29, 201)**	**166 (73, 261)**	**4 (3, 6)**	**14 (8, 22)**	6 (3, 8)	NA	5 (3, 7)	NA
Specimen collection to IgM testing	**9 (7, 13)**	**14 (9, 21)**	12 (7, 19)	13 (8, 25)	76 (57, 104)	72 (43, 103)	**12 (7, 23)**	**17 (8, 35)**	NA	NA	NA	NA
**Health region distribution, n (%)** ^**§**^												
Aguadilla	2 (0.2)	1821 (11.3)	0 (0)	174 (5.4)	2 (0.1)	140 (3.0)	0 (0)	1816 (5.2)	2 (0.4)	NA	2 (0.8)	NA
Arecibo	0 (0)	1854 (11.5)	2 (0.7)	287 (8.9)	4 (0.2)	469 (10.1)	2 (0.1)	4050 (11.6)	10 (1.8)	NA	0 (0)	NA
Bayamon	0 (0)	2376 (14.7)	20 (7.0)	811 (25.1)	5 (0.2)	238 (5.1)	5 (0.3)	8067 (23.1)	61 (11.2)	NA	23 (9.7)	NA
Caguas	15 (1.3)	1133 (7.0)	7 (2.5)	263 (8.1)	8 (0.3)	1115 (24.0)	6 (0.3)	2950 (8.4)	24 (4.4)	NA	5 (2.1)	NA
Fajardo	3 (0.3)	286 (1.8)	7 (2.5)	54 (1.7)	14 (0.6)	738 (15.9)	1 (0.1)	380 (1.1)	20 (3.7)	NA	2 (0.8)	NA
Mayaguez	6 (0.5)	2745 (17.0)	0 (0)	307 (9.5)	0 (0)	161 (3.5)	2 (0.1)	3450 (9.9)	0 (0)	NA	3 (1.3)	NA
San Juan	36 (3.2)	3849 (23.8)	196 (68.8)	1216 (37.6)	300 (13.0)	1739 (37.4)	14 (0.7)	6683 (19.1)	267 (49.1)	NA	58 (24.4)	NA
Ponce	1064 (94.5)	2010 (12.4)	52 (18.2)	110 (3.4)	1955 (84.9)	32 (0.7)	1885 (98.4)	7464 (21.3)	157 (28.9)	NA	144 (60.5)	NA
Unknown	0 (0)	90 (0.6)	1 (0.4)	12 (0.4)	15 (0.7)	18 (0.4)	1 (0.1)	135 (0.4)	3 (0.6)	NA	1 (0.4)	NA

DENV = dengue virus; CHIKV = chikungunya virus; ZIKV = Zika virus; SARS-CoV-2 = Severe acute respiratory syndrome coronavirus 2; SEDSS = Sentinel Enhanced Dengue Surveillance System; PADSS = Puerto Rico Department of Health Passive Disease Surveillance System; PCR = polymerase chain reaction; IgM = Immunoglobulin M; NA = not available; these data were not available to investigators; IQR = interquartile range

DENV epidemic period included positive tests from January 1, 2012, to December 31, 2014; DENV non-epidemic period: January 1, 2019, to December 31, 2023; CHIKV: May 28, 2014, to July 31, 2015; ZIKV: January 21, 2016, to March 23, 2017; COVID-19: pre-Omicron March 8, 2020, to November 27, 2021, Omicron November 28, 2021 to February 28, 2022.

Bolded values indicate comparisons with P < 0.01; Pearson chi-square tests were used for categorical variables and Wilcoxon rank-sum tests for timing variables. For health region distribution, significance reflects the overall comparison across all regions within each pathogen-period.

* Persons who underwent both PCR and IgM testing and had positive results were counted once in the total number of laboratory-confirmed cases; therefore, PCR and IgM categories are not mutually exclusive. The earliest specimen collection date was used.

† IgM testing was conducted using IgM antibody capture (MAC)-ELISA. During the epidemic period, any positive IgM result was considered positive; during the non-epidemic period, DENV IgM-positive results with negative ZIKV IgM were considered indicative of DENV infection.

‡ Timing variables are presented as median days (IQR).

§ Health region values are presented as n (%) of laboratory-confirmed cases within each surveillance system and pathogen-period column.

### Representativeness of dengue, chikungunya, Zika, and COVID-19 trends

**Fig 1:**
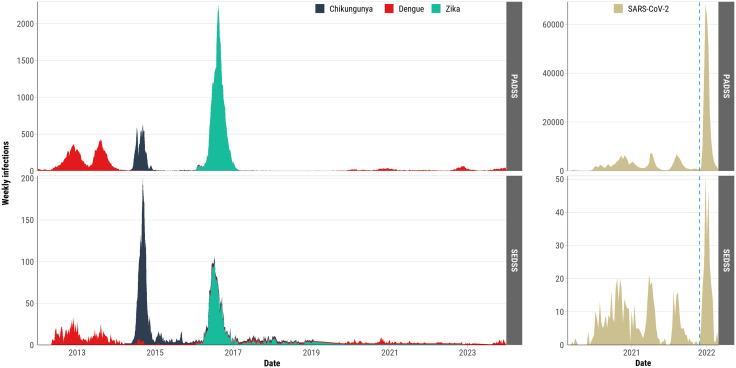
Weekly arbovirus and COVID-19 cases by symptom onset date and surveillance system type, Puerto Rico, 2012–2023. This figure compares cases reported by the Sentinel Enhanced Dengue Surveillance System (SEDSS) and the Puerto Rico Department of Health (PRDH) Passive Disease Surveillance System (PADSS). Arbovirus trends (left panels) include dengue, chikungunya, and Zika, while COVID-19 trends (right panels) are divided into pre-Omicron (before November 28, 2021) and Omicron (November 28, 2021, and after) periods. The dashed blue line indicates the beginning of the Omicron period.

**Fig 2:**
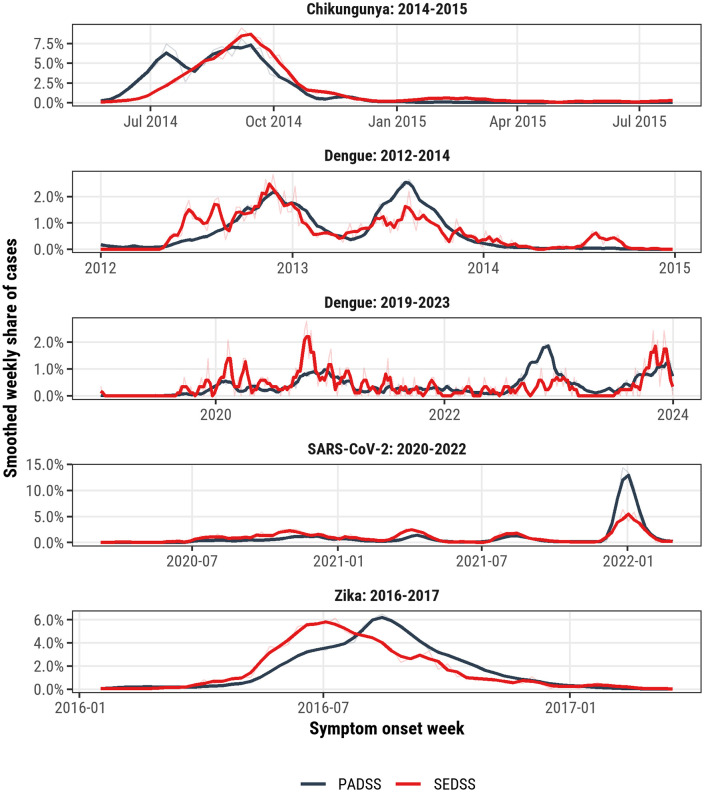
Standardized smoothed weekly trajectories of arbovirus and COVID-19 cases by symptom onset week and surveillance system, Puerto Rico, 2012–2023. Weekly case series from the Sentinel Enhanced Dengue Surveillance System (SEDSS) and the Puerto Rico Department of Health Passive Disease Surveillance System (PADSS) are shown for dengue (2012–2014 and 2019–2023), chikungunya (2014–2015), Zika (2016–2017), and SARS-CoV-2 (2020–2022). Faint lines represent the observed weekly share of cases, and darker lines represent the centered 3-week moving average. Smoothed counts were standardized within each pathogen-period and surveillance system and plotted as weekly share of cases to facilitate comparison of temporal pattern and timing rather than absolute case volume.

Observed weekly case counts (Fig 1) and standardized smoothed weekly case-share trajectories (Fig 2) for dengue, chikungunya, Zika, and COVID-19 in SEDSS and PADSS followed similar temporal distributions. The standardized smoothed trajectories show that Zika cases peaked earlier in SEDSS compared to PADSS.

### Correlation of lagged dengue, chikungunya, Zika, and COVID-19 SEDSS cases to PADSS

**Fig 3:**
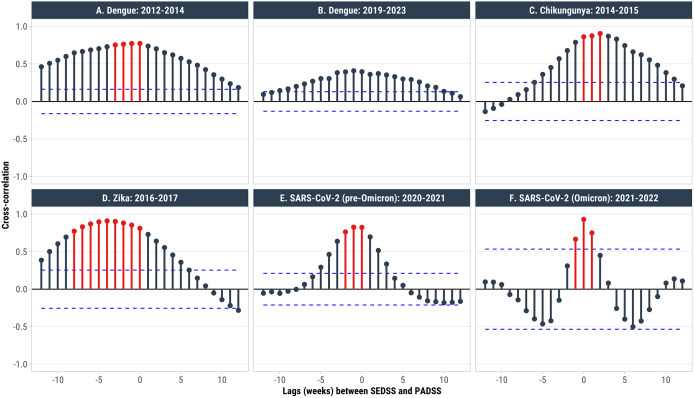
Cross-correlation coefficients (CCFs) comparing arboviral (panels A–D) and COVID-19 (panels E and F) cases reported by symptom onset date in SEDSS at lags of ±12 weeks relative to PADSS. CCFs were calculated from the raw weekly case series; no smoothing was applied before analysis. Blue dashed lines denote the significance threshold for individual CCFs. Red points and bars denote lag values with CCFs that were not statistically different from each other, indicating that CCFs at those lags were similar. For example, in Panel A, epidemic dengue cases reported in SEDSS up to a lag of -3 weeks are highly correlated with PADSS at week 0 (i.e., when public health authorities should be receiving information on cases). This indicates that reporting in SEDSS precedes PADSS by up to three weeks. The time periods were defined using the following dates. DENV epidemic period: January 1, 2012–December 31, 2014; DENV non-epidemic period: January 1, 2019–December 31, 2023; CHIKV: May 28, 2014–July 31, 2015; ZIKV: January 21, 2016–March 23, 2017; COVID-19: pre-Omicron March 8, 2020–November 27, 2021; Omicron: November 28, 2021–February 28, 2022.

During epidemic dengue the highest cross-correlation (0.77) was observed at a lag of 0 and -1 weeks. The CCF values at lags 0 to -3 weeks were not statistically different (with 95% confidence) from that at its peak of 0.77. Thus, the data are consistent with a CCF whose peak could occur at any lag from -3–0 weeks, inclusive, suggesting that SEDSS reporting precedes PADSS reporting by anywhere between 3 and 0 weeks (Fig 3, panel A). The CCF for non-epidemic dengue was generally low, indicating low temporal concordance between the two reporting systems (Fig 3, panel B). Among chikungunya cases, the highest value of the CCF (0.90) was observed at week +2, but CCFs at lags 0, + 1, and +2 were not statistically different (Fig 3, panel C), suggesting that SEDSS and PADSS detected increasing chikungunya trends at broadly similar times, with only limited evidence that PADSS may have captured rising transmission slightly earlier. The CCF for Zika was higher compared to other pathogens, reaching its highest value of 0.91 at -4 weeks. CCFs for lagged weekly Zika counts from 0 to -8 weeks were not significantly different, indicating that reported cases in SEDSS can precede reporting in PADSS by up to eight weeks. Among COVID-19 cases in the pre-Omicron period the highest CCF (0.82) occurred at lags of -1 and 0 weeks (Fig 3, panel E). CCFs at lags 0, -1, and -2 were not statistically different indicating that SEDSS cases may precede cases reported to PADSS by one or two weeks. In contrast, in the Omicron period, the highest CCF was observed at week 0 (0.93) and cross-correlations at lags –1, 0, and 1 were not statistically different from one another suggesting SEDSS cases were concordant but did not precede PADSS cases during the Omicron period.

## Discussion

A sentinel surveillance system in Puerto Rico provided representative island-wide estimates and could serve as an early warning system for emerging and epidemic pathogens. Visual comparisons of trends between the sentinel and passive surveillance systems revealed broadly similar trends for epidemic and non-epidemic dengue, chikungunya, Zika, and COVID-19. In addition, we observed that cases at sentinel sites could precede those in island-wide passive reporting by up to eight, three, and two weeks for Zika, epidemic dengue, and pre-Omicron COVID-19, respectively. Our findings highlight the utility of sentinel surveillance in resource-limited settings, providing representative and timely estimates of transmission for emerging and epidemic pathogens.

Our findings are supported by previous studies comparing passive, sentinel, and cohort-based surveillance for respiratory and vector-borne viruses. Studies comparing sentinel with passive or mandatory surveillance for influenza and measles have shown that sentinel systems can produce trends that are representative of broader population patterns and, in some settings, detect increases earlier than passive surveillance [29–32]. Few studies have directly compared dengue sentinel and passive surveillance systems. In Ecuador, a comparison of active cluster-based and passive surveillance found that dengue cases were identified 7 weeks earlier through active surveillance [18]. Our study builds on this literature by providing a direct comparison of sentinel and passive surveillance for dengue and other emerging pathogens in Puerto Rico.

Reporting in SEDSS did not significantly precede PADSS for non-epidemic dengue cases, and temporal concordance between the two systems was lower than during epidemic dengue. This likely reflects the more sporadic, focal, and geographically heterogeneous nature of dengue transmission during non-epidemic periods, when smaller weekly case counts and localized clusters can reduce week-to-week correlation between a sentinel system and island-wide passive surveillance even when broad temporal patterns remain similar. For chikungunya, PADSS may have captured increasing case trends slightly earlier than SEDSS; however, because CCFs at lags 0, + 1, and +2 were not statistically different, the two systems likely detected rising chikungunya activity at broadly similar times overall. This may reflect the fact that during 2014–2015 SEDSS was concentrated in the Ponce region, whereas PADSS captured cases island-wide, including the Metro San Juan Area and northeastern Puerto Rico, where chikungunya transmission was more intense.

Having a robust sentinel surveillance system in resource-limited settings has many advantages. First, SEDSS was able to rapidly implement testing for novel pathogens to detect ongoing circulation of Zika, chikungunya, and COVID-19. Despite significant delays during the chikungunya outbreak, PCR turnaround times were substantially shorter in SEDSS. Faster test implementation and turn-around times of specimens collected from a sentinel site during an outbreak of an emerging disease can enhance preparedness and monitoring of disease trends beyond the capabilities of a passive surveillance system. Second, standardized arboviral laboratory testing allowed differentiation of arboviral infections amidst overlapping signs and symptoms and co-circulation with respiratory viruses [33]. Through SEDSS, we confirmed minimal circulation of dengue during the chikungunya and Zika epidemics [34]. Third, during the COVID-19 pandemic, 59% of all PCR testing for dengue was performed in SEDSS, with <10% of participants testing positive, indicating low levels of dengue transmission that may have been overlooked if relying solely on the 30–60% test-positivity observed in the passive surveillance system. Finally, SEDSS allows for more precise denominators to measure hospitalization and complications of emerging viruses, such as chikungunya.

These findings also highlight the limitations of the sentinel surveillance approach. The first is that laboratory capacity can be rapidly overwhelmed in large epidemics. During the first year of the chikungunya epidemic, laboratory confirmation of chikungunya was limited to hospitalized cases captured by PADSS and SEDSS. This limited the utility of the sentinel surveillance approach by biasing testing to more severe cases and decreasing the detection of more common, mild cases in a timely manner. A second limitation is related to the geographical variability of where cases occur. During the chikungunya epidemic, SEDSS was limited to the Ponce region. Although Ponce had a high incidence of chikungunya cases, the highest incidence was seen in the Metro San Juan Area and the northeastern region of the island. Thus, having limited capture of Puerto Rico’s population likely resulted in no advantage in case detection earlier than PADSS. In contrast, a SEDSS recruitment site was present in the San Juan Metro Area during the COVID-19 pandemic which likely contributed to early warning by SEDSS relative to PADSS. Similarly, SEDSS captured relatively few non-epidemic dengue cases from some parts of the island, particularly western Puerto Rico in 2023, which may have contributed to the low CCFs observed between SEDSS and PADSS. During non-epidemic periods, dengue transmission is often more localized and sporadic than during major epidemics, so limited geographic coverage of sentinel sites may reduce temporal concordance with island-wide passive surveillance. Enhancements to the geographical representativeness of sentinel sites could improve the early warning and monitoring of emerging infections.

Implementing sentinel surveillance for emerging pathogens requires careful consideration of multiple factors. First, costs can be high compared to passive surveillance in single facilities. Yearly direct and indirect costs to maintain multiple SEDSS sites, including laboratory supplies, staff, and study spaces, among others, amount to approximately $700,000 US dollars. However, costs may be significantly lower in other settings, and the preparedness and research capabilities of sentinel surveillance sites can be leveraged to offset costs to the funding entity through partnerships with academic institutions and public health agencies. Second, human and infrastructure needs are high. SEDSS requires dedicated recruiters, data analysts, and study site coordinators, as well as access to a reference laboratory that can promptly run tests and report back to study sites. Third, size matters. Given Puerto Rico’s size, 1–2 sentinel sites may be sufficient, whereas appropriate coverage for larger geographical areas would likely require more, thereby increasing costs. Finally, sentinel sites have lower limits of sensitivity and upper limits of recruitment, which may affect the generalizability of findings in large epidemics. During the Omicron period, weekly SEDSS case counts did not exceed 53 cases, limiting the system’s ability to capture the magnitude of increased transmission during this period. Nevertheless, the benefits of early detection of transmission through sentinel surveillance, as observed in SEDSS, could significantly reduce morbidity, mortality, and economic costs that may offset initial investments [35]. The findings from SEDSS were also shared in weekly reports to the Puerto Rico Department of Health and used during outbreaks to provide situational awareness and guide response planning decisions.

Our study is subject to at least four limitations. First, sentinel sites changed over time and they were not dispersed evenly throughout Puerto Rico, which may have affected the representativeness and timeliness of our findings and the variability by pathogen. However, the addition of a sentinel site in the San Juan Metro Area in 2018 likely increased the ability of SEDSS to detect emerging pathogens because of its high population density and role as a port of entry to the island. Second, laboratory testing during the chikungunya outbreak of 2014–2015 was substantially delayed island-wide because of test availability. This likely affected timeliness in both surveillance systems and may have contributed to the small apparent lead of PADSS over SEDSS for chikungunya; therefore, the observed lag structure for chikungunya should be interpreted with caution. Third, SEDSS estimates are dependent on healthcare-seeking behavior. Participation in SEDSS is voluntary, and only those presenting to care can enroll. Fourth, relying primarily on hospital-based sentinel surveillance may have skewed our results, leading to under-capture of mild disease, although the comprehensive testing of all participants with fever could increase detection of dengue among patients with mild symptoms compared to surveillance systems where only hospitalized or severe cases are tested. Ensuring participation in outpatient clinics has been shown to provide better capture of mild-to-moderate disease during the COVID-19 pandemic and provide early warning relative to hospitalizations. Despite its limitations, our study’s strength stems from robust longitudinal data spanning multiple emerging pathogen epidemics and examination of different transmission periods for endemic dengue.

In summary, a sentinel surveillance system in Puerto Rico provided representative estimates of pathogen transmission and could serve as an early warning system for dengue epidemics and emerging pathogens such as Zika and SARS-CoV-2. Implementing sentinel surveillance in a low-resource setting should weigh the benefits of early detection of emerging pathogens against the higher cost of surveillance. Additionally, the ability to implement rapid public health action should be considered to determine whether this type of surveillance suits the context and needs.
